# Quality of INR control and switching to non-Vitamin K oral anticoagulants between women and men with atrial fibrillation treated with Vitamin K Antagonists in Spain. A population-based, real-world study

**DOI:** 10.1371/journal.pone.0211681

**Published:** 2019-02-12

**Authors:** Aníbal García-Sempere, Isabel Hurtado, Daniel Bejarano-Quisoboni, Clara Rodríguez-Bernal, Yared Santa-Ana, Salvador Peiró, Gabriel Sanfélix-Gimeno

**Affiliations:** Health Services Research Unit, Foundation for Biomedical Research of Valencia—FISABIO, Valencia, Spain; Leiden University Medical Center, NETHERLANDS

## Abstract

**Background:**

Worldwide, there is growing evidence that quality of international normalized ratio (INR) control in atrial fibrillation patients treated with Vitamin K Antagonists (VKA) is suboptimal. However, sex disparities in population-based real-world settings have been scarcely studied, as well as patterns of switching to second-line Non-VKA oral anticoagulants (NOAC). We aimed to assess the quality of INR control in atrial fibrillation patients treated with VKA in the region of Valencia, Spain, for the whole population and differencing by sex, and to identify factors associated with poor control. We also quantified switching to Non-VKA oral anticoagulants (NOAC) and we identified factors associated to switching.

**Methods:**

This is a cross-sectional, population-based study. Information was obtained through linking different regional electronic databases. Outcome measures were Time in Therapeutic Range (TTR) and percentage of INR determinations in range (PINRR) in 2015, and percentage of switching to NOAC in 2016, for the whole population and stratified by sex.

**Results:**

We included 22,629 patients, 50.4% were women. Mean TTR was 62.3% for women and 63.7% for men, and PINNR was 58.3% for women and 60.1% for men (p<0.001). Considering the TTR<65% threshold, 53% of women and 49.3% of men had poor anticoagulation control (p<0.001). Women, long-term users antiplatelet users, and patients with comorbidities, visits to Emergency Department and use of alcohol were more likely to present poor INR control. 5.4% of poorly controlled patients during 2015 switched to a NOAC throughout 2016, with no sex differences.

**Conclusion:**

The quality of INR control of all AF patients treated with VKA in 2015 in our Southern European region was suboptimal, and women were at a higher risk of poor INR control. This reflects sex disparities in care, and programs for improving the quality of oral anticoagulation should incorporate the gender perspective. Clinical inertia may be lying behind the observed low rates of switching in patient with poor INR control.

## Introduction

Patients with atrial fibrillation (AF) are at an increased risk of stroke and thus require anticoagulant prophylaxis. For decades, treatment with vitamin K antagonists (VKA) has been the gold standard for stroke prevention in AF [1]. The use of oral anticoagulants such as warfarin has been shown in clinical trials to reduce the risk of stroke by two thirds [2]. However, the efficacy and safety of VKA are closely associated with the quality of anticoagulation control. Use of VKA can be challenging due to their narrow therapeutic range, as therapy must be tightly controlled and maintained within a therapeutic index of international normalized ratio (INR) values of between 2 and 3. Additionally, the need for periodic INR monitoring, high inter-patient variability in treatment response, numerous drug and food interactions and medication non-adherence are well-documented barriers to optimal INR control [3–9].

There is a growing body of evidence showing that INR control in routine clinical practice, and even in clinical trials, is usually far from ideal, close to poor and even patient-endangering. Many registry-based studies, real-world studies and systematic reviews have consistently reported that INR control in routine clinical practice is largely suboptimal [10–18]. Time in Therapeutic Range (TTR), the more commonly used measure of anticoagulation control expressing the percentage of time a patient is correctly anticoagulated with INR values of between 2 and 3, shows wide variations depending on settings, organizations and patients [19]. Also differing calculation methods for TTR and thresholds for the definition of “good control” are used, varying within organisations and over time. For instance, TTR≥70% is defined as optimal care by the European Society of Cardiology (ESC), whether a TTR<65% is defined as suboptimal care by the National Institute of Clinical Excellence (NICE) [8], and recent evidence suggests the threshold of good control should be elevated to >80% to minimize risks [20]. All in all, evidence worldwide shows that a large proportion of VKA treated patients, ranging from one third to three quarters, do not achieve adequate INR control and are thus at an increased risk of stroke (when INR<2) or bleeding (when INR>3). Furthermore, sex (being a woman) has been identified as an independent predictor of poor TTR [21], but the extent of differences between women and men has not to date been quantified in a real-world setting.

In the Spanish NHS with universal healthcare coverage, evidence on INR control quality is in line with that observed abroad, showing that poor INR control may be affecting between one and two thirds of patients using VKA. However, studies addressing this issue are sparse and based on collaborative research registries or in local healthcare centres with reduced populations [22–30], with absence of studies based on information routinely collected from the entire population served, and thus the generalizability of their results may be limited or they may not accurately reflect average ordinary clinical practice. Additionally, these studies systematically ignore the sex perspective. Also, patterns of switching from VKA to Non-VKA Oral Anticoagulants (NOAC) are unknown, although NOAC are relegated to a second line of treatment after VKA in Spain. NOAC use in Spain is subject to conditions such as poor INR control, ineffectiveness of or contraindication to VKA, increased risk of intracranial hemorrhage or inability to access INR facilities. This study aims to assess the quality of INR control per sex in 2015 in the whole population of patients treated with acenocoumarol for AF in the region of Valencia, and to identify factors associated with poor INR control. We further aimed to describe patterns of switching from VKA to NOAC during 2016 and to identify factors associated to switching patterns. Main analyses are performed for the whole population and stratified by sex.

## Methods

### Design and setting

This cross-sectional population-based study was conducted in the Valencia Health Agency (VHA), the public health system of the region of Valencia in Spain, covering about 97% of the 5 million inhabitants region's population. We selected all patients diagnosed AF or flutter [diagnosis code of International Classification of Diseases, Ninth Revision, Clinical Modification (ICD-9-CM) 427.31 and 427.32] treated with acenocoumarol in 2015 (marginal use of warfarin, phenprocoumon or fluindione, mainly by non-residents, was not included).

**Fig 1:**
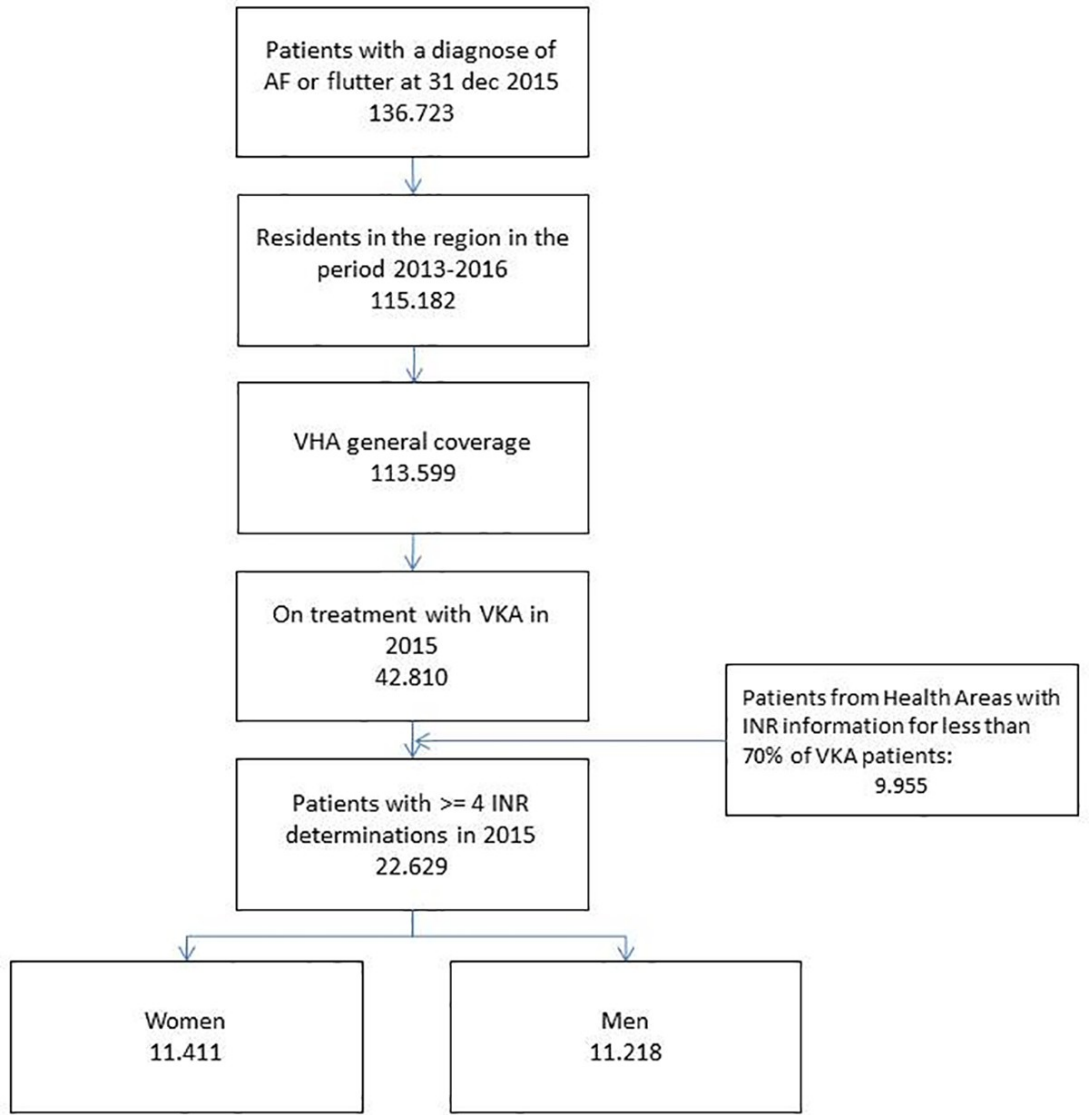
Flowchart.

We defined patients treated with acenocoumarol in 2015 by those having at least one dispensation of the drug in the last quarter of 2015, by having initiated acenocoumarol before December 2014 and by not having any prescription for any other oral anticoagulants in 2015. Additionally, we only selected patients with at least 4 INR determinations in 2015. People without pharmaceutical/health coverage by the VHA, mainly some government employees whose prescriptions are reimbursed by civil service insurers and thus not included in the pharmacy databases of the VHA, and patients not registered in the municipal census (non-residents or temporary residents), or those who left the region or were disenrolled from VHA coverage for other causes, were excluded because of limitations on follow-up. Additionally, availability of information about INR determinations in the EMR was not homogeneous for each of the 24 Health Areas (HAs, the administrative and territorial management units in the region) that make up the public health care provision network in the region. INR data is linked to the EMR from local, HA-based INR records, and this process has been implemented in a disparate manner by HAs. We only include patients belonging to HAs with INR information for at least 70% of their patients (8 HAs were excluded, representing only 23% of patients; see Fig 1 and S1 Table).

### Data sources

Information was obtained from the VHA electronic information systems. The Population Information System (SIP) provides information on the population under VHA coverage and registers certain demographic characteristics, including the geographical location and contextual situation of each person and dates and causes of VHA discharge, including death. The Minimum Basic Dataset (MBDS) at hospital discharge is a synopsis of clinical and administrative information on all hospital discharges, including diagnoses and procedures (ICD codes). The electronic medical record for ambulatory care (EMR), available in all primary healthcare centers and walk-in facilities, has information about diagnoses, personal, and family medical history, laboratory results and lifestyle as well as information about both physician prescriptions and dispensations from pharmacy claims. Pharmaceutical prescription and dispensation data, including concomitant medication, is highly reliable as it used for reimbursement purposes. INR information in the EMR is retrieved from HA-based INR records registered by hematologists and primary care doctors who manage oral anticoagulation in each HA. All the information in these systems is linked at an individual level through a unique identifier.

### Outcome measures

Main outcome measures were the Time in Therapeutic Range (TTR) using the Rosendaal linear interpolation method and the percentage of INR determinations in range (PINRR). We calculated TTR and PINRR using all INR determinations available throughout the whole year 2015. We also calculated the percentage of switching from acenocoumarol to direct oral anticoagulants (NOACs: apixaban, dabigatran or rivaroxaban) in 2016.

### Covariates

Variables potentially related to the risk of atrial fibrillation and to the use of oral anticoagulants in the study population over the study period were considered. These included socio-demographic characteristics, comorbidities and healthcare resource utilisation in the preceding 12 months. Based on comorbidity information, we calculated and added relevant patient-level risk predictor scores—CHADS2, CHA2DS2-VASC, and HAS-BLED scores–to the dataset.

### Analysis

First, we described patient characteristics. Second, we assessed the quality of INR control by calculating TTR (time in therapeutic range) using Rosendaal and PINRR, and we obtained the percentage of patients with poor control, using two updated and relevant definitions of poor control: the commonly used threshold of TTR<65% (and recommended by the UK’s NICE) and the threshold proposed by the ESC of TTR<70%. Third, to identify factors associated with poor INR control we used multivariable regression analysis. Fourth, we described the patterns of switching from acenocoumarol to NOAC in the following year, 2016. Fifth, we again used logistic regression analysis, including a dichotomous variable of INR control, to identify factors associated with switching to NOAC (estimates were calculated using the Rosendaal method and the NICE threshold). We used stepwise regression models with entry and exit significance levels of 0.05 and 0.1, respectively. We carried out additional sensitivity analyses with regard to acceptable INR ranges of [1.8–3.2] instead of [2–3], as some studies employ this measure justified the potential margin of error of the coagulometer and real-world reluctance to modify treatment in face of slight INR deviations [24, 31, 32] (± 0.2). C-Statistics was used to assess model discrimination and Hosmer-Lemeshow test for calibration. Finally, we compared our selected population to the whole number of AF patients treated with acenocoumarol in the region in 2015 to check for the generalizability of our results. All calculations and statistical analyses were conducted using STATA 14 (StataCorp, College Station, TX).

### Ethics

The study protocol was approved by the regional Ethics Committee for Clinical Research of the General Directorate of Public Health and the Centre for Public Health Research. Informed patient consent was waived because, according to European rules and the Spanish laws on data privacy, the Valencia Government Health Department transferred to researchers only non-identifiable data.

## Results

### Patient characteristics

A total of 22,629 AF patients treated with acenocoumarol with at least 4 INR determinations in the year 2015 and meeting inclusion and exclusion criteria were included in the study (Fig 1). Mean age was 77 years old, 50.4% were women, 81.5% had hypertension, 14.8% had had a previous stroke or TIA and 45.2% were long-term acenocoumarol users (patients using acenocoumarol for more than 6 years). Mean number of INR determinations during 2015 was 14 (median: 13; p25: 10; p75: 17), and 95.3% of patients had a CHA2DS2-VASC score ≥2 and 87.1% a HAS-BLED score ≥3.

Women were older (mean age was 78.1 vs 75.6 in men, p<0.001, and 71.8% aged 75 and over vs 59.9% for men), more deprived (89.2% earning less than 18.000 euros/year vs 80.2%; and 6.3% were at risk of social exclusion, compared to 2.8% of men), had more comorbidities such as prior congestive heart failure, hypertension, thromboembolism, dementia or depression, and presented higher stroke and bleeding risks scores. Men had more prior vascular disease and gastrointestinal bleeding (22.8% vs 14.3% in women and 7.8% vs 6.7% in women, respectively), malignancy and alcohol use, and also used more antiplatelet medication (20.7% vs 13.8% in women). No sex differences were found with regard to time in treatment with AVK, renal disease, hemorrhagic stroke or use of NSAIDs (Table 1).

**Table 1:** Patient characteristics, by sex and for the whole cohort.

	Total	Female	Male	p-value
**N**	22,629	11,411 (50.43%)	11,218 (49.57%)	
**Age**				<0.001
**< 65**	2,132 (9.42%)	799 (7.00%)	1,333 (11.88%)	
**65–74**	5,589 (24.70%)	2,421 (21.22%)	3,168 (28.24%)	
**≥75**	14,908 (65.88%)	8,191 (71.78%)	6,717 (59.88%)	
**Country**				<0.001
**ESP**	21,163 (93.52%)	10,766 (94.35%)	10,397 (92.68%)	
**EUR**	686 (3.03%)	260 (2.28%)	426 (3.80%)	
**NON-EUR**	272 (1.20%)	136 (1.19%)	136 (1.21%)	
**DES**	508 (2.24%)	249 (2.18%)	259 (2.31%)	
**Income**				<0.001
**0–18.000**	19,181 (84.76%)	10,182 (89.23%)	8,999 (80.22%)	
**> 18.000**	3,448 (15.24%)	1,229 (10.77%)	2,219 (19.78%)	
**Risk of social exclusion**	1,035 (4.57%)	724 (6.34%)	311 (2.77%)	<0.001
***Diagnosis***				*<0*.*001*
**Atrial fibrillation**	21,624 (95.56%)	11,030 (96.66%)	10,594 (94.44%)	
**Flutter**	1,005 (4.44%)	381 (3.34%)	624 (5.56%)	
**Time since Therapy Initiation**				0.703
**1–3 Years**	5,411 (23.91%)	2,739 (24.00%)	2,672 (23.82%)	
**3–6 Years**	6,611 (29.21%)	3,305 (28.96%)	3,306 (29.47%)	
**> 6 Years**	10,607 (46.87%)	5,367 (47.03%)	5,240 (46.71%)	
***Comorbidities***				
**Congestive heart failure**	4,759 (21.03%)	2,693 (23.60%)	2,066 (18.42%)	<0.001
**Hypertension**	18,817 (83.15%)	9,677 (84.80%)	9,140 (81.48%)	<0.001
**Diabetes**	8,905 (39.35%)	4,342 (38.05%)	4,563 (40.68%)	<0.001
**Liver disease**	2,095 (9.26%)	1,017 (8.91%)	1,078 (9.61%)	0.070
**Renal disease**	3,684 (16.28%)	1,879 (16.47%)	1,805 (16.09%)	0.443
**Previous ischemic stroke or TIA**	3,241 (14.32%)	1,664 (14.58%)	1,577 (14.06%)	0.260
**Thromboembolism**	1,609 (7.11%)	974 (8.54%)	635 (5.66%)	<0.001
**Hemorrhagic stroke**	160 (0.71%)	77 (0.67%)	83 (0.74%)	0.559
**Gastrointestinal bleeding**	1,644 (7.27%)	767 (6.72%)	877 (7.82%)	0.001
**Other bleeding**	7,596 (33.57%)	4,009 (35.13%)	3,587 (31.98%)	<0.001
**Vascular disease**	4,191 (18.52%)	1,636 (14.34%)	2,555 (22.78%)	<0.001
**Dementia**	1,916 (8.47%)	1,156 (10.13%)	760 (6.77%)	<0.001
**Depression**	3,403 (15.04%)	2,460 (21.56%)	943 (8.41%)	<0.001
**Cancer**	3,878 (17.14%)	1,570 (13.76%)	2,308 (20.57%)	<0.001
**Alcohol**	189 (0.84%)	12 (0.11%)	177 (1.58%)	<0.001
***Healthcare utilisation (mean*, *SD)***				
**Hospitalizations**	0.54 (1.16)	0.50 (1.14)	0.56 (1.18)	<0.001
**ED visits**	1.00 (2.00)	1.06 (2.08)	0.94 (1.91)	<0.001
**Outpatients visits**	12.13 (7.66)	12.84 (7.86)	11.40 (7.38)	<0.001
**Specialist visits**	3.22 (4.64)	3.09 (4.57)	3.34 (4.71)	<0.001
**Cardiology visits**	0.83 (1.18)	0.79 (1.13)	0.88 (1.23)	<0.001
**Neurologic visits**	0.17 (0.60)	0.17 (0.60)	0.16 (0.59)	0.367
**Mental Health visits**	0.11 (0.89)	0.14 (0.93)	0.08 (0.86)	<0.001
**Social care visits**	0.11 (0.74)	0.12 (0.80)	0.09 (0.67)	0.004
***Medication use***				
**NSAID**	2,328 (10.29%)	1,202 (10.53%)	1,126 (10.04%)	0.219
**Antiplatelet**	1,903 (8.41%)	593 (5.20%)	1,310 (11.68%)	<0.001
***Scores***				
**CHADS2 score ≥2**	17,495 (77.31%)	9,155 (80.23%)	8,340 (74.34%)	<0.001
**CHA2DS2-VASC score ≥ 2**	21,567 (95.31%)	11,231 (98.42%)	10,336 (92.14%)	<0.001
**HAS-BLED ≥2**	22,238 (98.27%)	11,244 (98.54%)	10,994 (98.00%)	0.002
**HAS-BLED ≥3**	19,707 (87.09%)	10,170 (89.12%)	9,537 (85.02%)	<0.001

ESP: Spain; EUR: European; NON-EUR: Non-european; DES: Unknown; TIA: transient ischemic attack; ED: emergency department; NSAID: nonsteroidal anti-inflammatory drug.

### Quality of INR control

Mean TTR was 63% (62.3% for women and 63.7% for men, p<0.001), and PINNR was 59.2% (58.3% for women and 60.1% for men, p<0.001). Considering the TTR<65% threshold, 53% of women and 49.3% of men had poor anticoagulation control (p<0.001), rising to 63.2% and 60% respectively (p<0.001), when using the TTR<70% threshold. In sensitivity analysis, when using [1.8–3.2] as acceptable INR ranges and TTR<65% threshold for poor control, TTR rose to 75.5% for women and 76.8% for men (p<0.001), and PINNR was 72% and 73.6% for women and men (p<0.001), respectively; poor control affected from 22.5% to 30.8% of patients, depending on the threshold considered (Table 2).

**Table 2:** Mean TTR, PINRR and % of patients poorly controlled considering NICE (TTR≥65%) and ESC (TTR≥70%) thresholds and different acceptable INR range definitions.

	Total	Women	Men	p-value
Mean TTR and PINNR (Mean, SD)				
*INR range 2–3*				
TTR	63.0 (19.75)	62.3 (19.71)	63.7 (19.78)	<0.001
PINRR	59.2 (18.87)	58.3 (18.81)	60.1 (18.89)	<0.001
*INR 1*.*8–3*.*2*				
TTR	76.2 (17.94)	75.5 (17.96)	76.8 (17.90)	<0.001
PINRR	72.8 (17.45)	72.0 (17.51)	73.6 (17.34)	<0.001
*% patients poorly controlled (INR range 2–3)*				
**TTR<65%**				
TTR	11,579 (51.2%)	6,044 (53%)	5,535 (49.3%)	<0.001
PINRR	14,058 (62.1%)	7,338 (64.3%)	6,720 (59.9%)	<0.001
**TTR< 70%**				
TTR	13,950 (61.7%)	7,211 (63.2%)	6,739 (60.1%)	<0.001
PINRR	15,950 (70.5%)	8,252 (72.3%)	7,698 (68.6%)	<0.001
*% patients poorly controlled (INR range 1*.*8–3*.*2)*				
**TTR< 65%**				
TTR	5,096 (22.5%)	2,675 (23.4%)	2,421 (21.6%)	0.001
PINRR	6,928 (30.6%)	3,716 (32.6%)	3,212 (28.6%)	<0.001
**TTR< 70%**				
TTR	6,965 (30.8%)	3,655 (32.0%)	3,310 (29.5%)	<0.001
PINRR	8,951 (39.6%)	4,736 (41.5%)	4,215 (37.6%)	<0.001

TTR: Time in Therapeutic Range; PINNR: Percentage of INR determinations in Range; INR: International Normalized Ratio; INR: International Normalized Ratio.

Women, long-term acenocoumarol users, antiplatelet users and “high risk” patients (defined as patients with comorbidities such as heart failure, diabetes, depression, dementia, vascular disease, use of alcohol and ED visits) were more likely to present poor INR control. Higher income, age (being 65 years old and over), and visiting a neurologist or a cardiologist were associated with achieving good INR control (Table 3), but the predictive capacity of the model was low (C Statistics: 0.579).

**Table 3:** Factors associated with poor INR control.

	Odds Ratio	95%CI	p-value
*Socio-demographics*			
Female	1.13	1.07; 1.20	<0.001
Age 65–75 (ref: age<65)	0.88	0.80; 0.97	0.010
Age 75 and over (ref: age<65)	0.87	0.80; 0.95	0.004
Europe (country) (ref: Spain)	1.23	1.05; 1.44	0.007
Income >18.000e (ref: income ≤18.000)	0.89	0.82; 0.96	0.002
*Comorbidities*			
Congestive heart failure	1.19	1.12; 1.29	<0.001
Diabetes	1.14	1.08; 1.20	<0.001
Other bleeding	1.08	1.02; 1.14	0.011
Vascular disease	1.08	1.00; 1.16	0.036
Dementia	1.21	1.10; 1.35	<0.001
Depression	1.12	1.03; 1.20	0.005
Alcohol	1.70	1.25; 2.33	0.001
*Healthcare utilisation*			
Time since Therapy Initiation >6 years	1.05	1.00; 1.11	0.047
ED visits	1.04	1.03; 1.06	<0.001
Outpatient visits	1.01	1.00; 1.01	<0.001
Specialist visits	1.02	1.01; 1.03	<0.001
Cardiology visits	0.96	0.93; 0.99	0.012
Neurologic visits	0.91	0.86; 0.95	<0.001
Social care visits	1.04	1.00; 1.09	0.017
Antiplatelet	1.11	1.00; 1.23	0.045

n = 22629; LL: -15461.213; p: <0.001; r2: 0.014; C Statistic: 0.579; p (X2 Hosmer-Lemeshow): 0.807.Age (<65, 65–75, >75) and Country (Spain, Europe, Non-Europe, Unknown) are categorical variables. Sex, income, comorbidity variables and Time since Therapy Initiation >6 years are dichotomous variables. Visits are quantitative variables (the variable is number of visits), and accordingly the Odds ratios refer to the odds of presenting a poor INR control with every additional visit.

### Switching to NOAC

**Fig 2:**
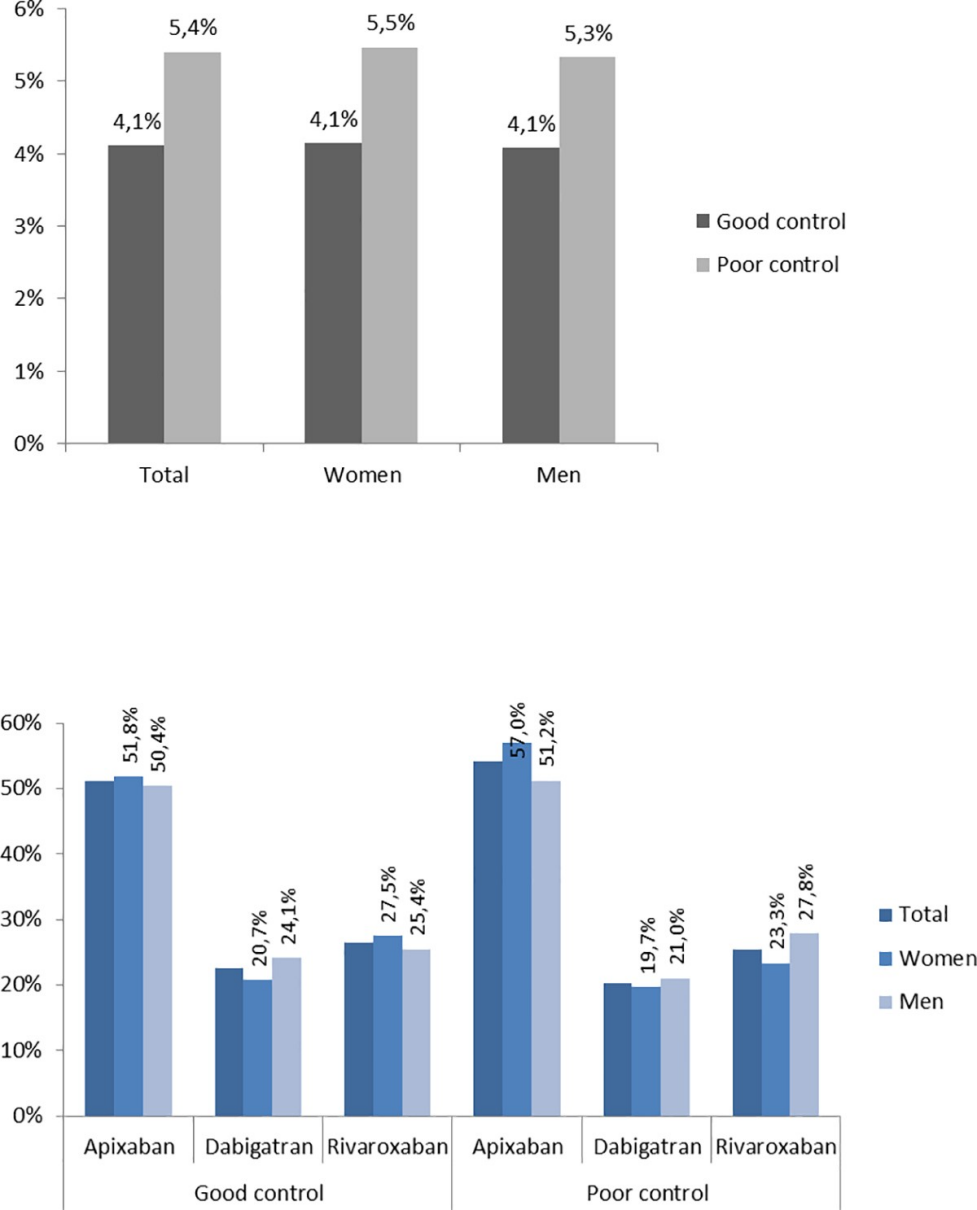
Percentage of switching to NOAC in 2016 by sex and quality of INR control, using Rosendaal’s TTR and TTR≥65% threshold (2a) and percentage of switching to the different NOACS in 2016 by sex and quality of INR control, using Rosendaal’s TTR and TTR≥65% threshold (2b).

Using Rosendaal’s TTR and the ≥65% threshold, 5.4% of poorly controlled patients during 2015 (5.5% women; 5.3% men) switched to a NOAC throughout 2016, as did 4.1% of patients with good INR control (similar for women and men), with similar figures when using the ≥70% threshold. From total switchers, and when considering the TTR≥65% threshold, 54.2% of poorly controlled and 51.1% of adequately controlled switched to apixaban in 2016, 25.4% and 26.4% to rivaroxaban, and 20.3% and 22.5% to dabigatran. No differences in terms of switching between women and men were found. Adequate INR control, presence of renal disease, and long-term use of acenocoumarol were associated with less likelihood of switching. Being non-European, having a higher income, more cardiology and primary care visits, and presence of vascular disease were positively associated with switching (Fig 2, Table 4). Predictive capacity of the model was also low (C-Statistics = 0.584).

**Table 4:** Factors associated with switching to NOAC.

	Odds Ratio	95%CI	p-value
*Socio-demographics*			
Non-Europe (country) (ref: Spain)	1,70	1.08;2.67	0.021
Income >18.000e (ref: income ≤18.000)	1,27	1.08;1.49	0.003
*Adequate INR control*	0.76	0.67;0.86	0.001
*Comorbidities*			
Renal disease	0.69	0.57; 0.83	0.001
Vascular disease	1.34	1.15;1.55	0.001
*Healthcare utilisation*			
Primary Care visits	1.01	1.00; 1.02	0.037
Cardiology visits	1.06	1.01; 1.11	0.018
Time since Therapy initiation>6 years	0.79	0.70; 0.89	0.001

n = 22629; LL: -4282.49; p: <0.0001; r2: 0.012; C Statistic: 0.59; p (X2 Hosmer Lemeshow): 0.573. Adequate INR control: TTR≥65% (ref: TTR<65%). Country (Spain, Europe, Non-Europe, Unknown) is a categorical variable. Income, comorbidity variables and Time since Therapy Initiation >6 years are dichotomous variables. Visits are quantitative variables (the variable is number of visits), and accordingly the Odds ratios refer to the odds of presenting a poor INR control with every additional visit.

## Discussion

In this real-world, population-based study, we show that the quality of INR control in AF patients treated with VKA in 2015 in the region of Valencia is suboptimal, and that women are at a higher risk of uncontrolled INR. Depending on the definition used for acceptable INR ranges and TTR threshold, a quarter to two-thirds of patients had inadequate INR control during 2015. We also found that switching to NOAC in the following year was as low as 5.4% for patients with inadequate control and 4.1% for patients with adequate INR control. Importantly, women had a worse mean TTR, PINRR and poorer INR control than men, irrespective of definitions. In fact, being a woman, using VKA for more than 6 years and being at high risk were factors associated with poor INR control, while wealthier, older patients and those visiting a cardiologist or neurologist were more prone to good INR control. These figures are especially noticeable as VKA involve around two thirds of OAC treatments for AF patients and around 50% of new treatments [9].

Figures on poor INR controlled patients switching to NOAC seem to be low, especially when poor INR control is established by national guidelines as a principal driver to switching to NOAC therapy. This may be revealing a problem of clinical inertia, but this finding should be interpreted with caution, as our design excludes patients who had switched to NOAC before 2016. This would also come to explain the finding that long-term use of VKA is associated with less likelihood of switching (as we are analyzing patients that somehow may be resistant to switching). No sex differences were found with regard to switching. Considering that women have worse INR control, a relative worst care and a stronger clinical inertia for women versus men could be inferred.

The proportion of patients with poor INR control change depending on the threshold for good INR control used. The threshold suggested by the ESC is more restrictive than the NICE threshold, which is in fact the one considered by the Spanish national rules. Roughly 10% of patients comprise between 65% and 70% of TTR, so in a context where NOACs are placed as second-line therapies and where poor INR control is a major reason for switching to NOAC [8], the decision to adopt one or another threshold could theoretically have a significant impact on practice. However, in the light of our results with regard to switching and additional past findings about initiation with NOAC [9], factors other than TTR thresholds seem to be driving NOAC prescription.

Sensitivity analyses with regard acceptable INR ranges result in significant variations in our estimates of the quality of INR control. The rationale used by other authors to employ INR ranges of [1,8–3,2] to estimate TTR is to account for potential coagulometer error and to avoid problems inherent to overcorrection [24, 31, 32]. However, these arguments are debatable, and the widely accepted and evidence-based INR range of [2–3] [33–40], which in fact is a simplification of the original threshold of [1,45–2,8] on which current anticoagulation clinical guidelines are still based [41–43], seems more appropriate for the purposes of assessment and comparison.

To the best of our knowledge, this is the first real-world data study that quantifies the differences in the quality of anticoagulation between women and men. Studies in experimental settings, registries or based on small populations [44–47] have also shown sex differences with female patients being more vulnerable overall than male patients, being older and more deprived, and results in terms of TTR and percentage of patients with good TTR being worse in every scenario. This calls for a redefinition of strategies for improving the management of VKA patients, where the gender gradient should be explicitly addressed at every stage as an essential driver for action. We further identified factors associated with INR control and switching. This information may be valuable to identify priority interventions for most vulnerable patients, and also to tackle the issue of therapeutic inertia in the case of inadequately controlled VKA patients. Finally, we confirm that our results in real-life patients from a Southern European region are similar to those of other real-world patients from very distinct settings, registry-based studies or clinical trials, and that operational definitions such as acceptable INR ranges or thresholds of good INR control may have a significant impact on the direction of results.

### Limitations

Our study is subject to some limitations. First, we included patients with at least 4 INR informed determinations in 2015. This excluded from analysis 47% of the total number patients treated with VKA in the region this year, raising a potential concern about the representativeness of our sample. However, we compared both populations (total VKA patients versus patients analyzed) and we found barely any differences (see Fig 1 and S1 Table).

Second, our study is cross-sectional in design. This allows for an accurate description of the “state of the art” of the quality of INR control in all patients treated with VKA in one moment of time (December 2015), but the interpretation of some of our results, especially with regard to patterns of switching, should be interpreted with caution. Our population may be somehow “resistant” to switching because include long-term users that remain under treatment after many years (and sometimes irrespective of their INR control). This may be lying behind the association identified between long-term use of VKA and poor INR control and less likelihood of switching [48], and also would explain the counterintuitive association of long-term VKA use with poor INR control. This would also explain, to some extent at least, the low rates of switching to NOAC observed in patients with uncontrolled INR. However, this information is still valuable because studies on INR control (commonly based on naïve users, as longitudinal follow-up of new users is a better design for inferring associations between exposure and outcomes) do not offer a view of the management of all the VKA patients in a moment of time, which is our goal in this study, and also because we bring the first population-based piece of evidence with regard to switching from VKA to NOAC in Spain. In a forthcoming study, we will evaluate a cohort of new VKA users and we will re-analyze the quality of INR control and switching, and we will check for consistency of our present estimates.

Third, despite including many relevant individual variables in our analysis, we cannot rule out the existence of omitted relative access to INR control facilities, or regarding the presence of a contraindication to NOAC, as these data are not routinely recorded in linkable clinical databases. These factors could be affecting some of our estimates, and further research should examine their influence on the quality of care, though their absence does not affect the relevance of our results. Fourth, information biases due to absent registration or differing data recording practices in the electronic databases might exist, although this is an inherent problem of any study using data from routine clinical practice. Moreover, misclassification (on exposure and covariates) is expected to be non-differential across groups of study subjects.

Fifth, although relevant predictors of poor INR control and clinical inertia have been identified, the discriminatory capacity of the regression model is low in both, suggesting that other non-identified factors are driving these phenomena. Sixth, we did not assess clinical outcomes, typically the occurrence of ischemic stroke, intracranial bleeding and other bleedings (including gastrointestinal bleedings) related to the quality of INR control, and we could now answer the question of to what extent differences in INR control among women and men translate into worse outcomes. We will perform this analysis in a cohort of new VKA users as this design is more suitable for inferring causal relationships between treatment and outcomes.

## Conclusion

This is the first study in our context to assess the quality of oral anticoagulation with VKA and switching to NOAC in AF patients on a population-basis using real-world data. The quality of INR control of all AF patients treated with VKA for stroke prevention in 2015 in our region was suboptimal, and women were at a higher risk of poor INR control. This reflects sex disparities in care, and programs for improving the quality of oral anticoagulation should incorporate the gender perspective at every step. In this sense, the approach used in our study with data from routine care could be incorporated into the EMR to improve patient follow-up. Observed low rates of switching in poor controlled patients is worrying, suggesting strong clinical inertia. Further studies should confirm our results, especially with regard to switching in new VKA users, and evaluate clinical outcomes associated with keeping patients with poor INR control on acenocoumarol.

## Supporting information

S1 Table Comparison of our population for analysis (VKA patients with at least 4 INR determinations in 2015) versus the whole population of VKA treated patients in 2015. (DOCX)
