# Supplementary material for: Quality of INR control and switching to non-Vitamin K oral anticoagulants between women and men with atrial fibrillation treated with Vitamin K Antagonists in Spain. A population-based, real-world study
Source: PLoS One. 2019 Feb 12;14(2):e0211681. doi: 10.1371/journal.pone.0211681 (PMC6372152; doi:10.1371/journal.pone.0211681)
Supplement: S1 Table — (DOCX) [file pone.0211681.s001.docx]

**S1 Table. Comparison of our population for analysis (VKA patients with at least 4 INR determinations in 2015) versus the whole population of VKA treated patients in 2015.**

|  | Total | Analysis population | VKA patients in 2015 | p |
| --- | --- | --- | --- | --- |
|  |  |  |  |  |
| N |  | 22,629 | 42,810 |  |
| Female | 32,765 (50.07%) | 11,411 (50.43%) | 21,354 (49.88%) | 0.184 |
| Age |  |  |  | 0.128 |
| < 65 | 6,336 (9.68%) | 2,132 (9.42%) | 4,204 (9.82%) |  |
| 65 – 74 | 16,294 (24.90%) | 5,589 (24.70%) | 10,705 (25.01%) |  |
| >75 | 42,809 (65.42%) | 14,908 (65.88%) | 27,901 (65.17%) |  |
| Country |  |  |  | 0.000 |
| ESP | 61,216 (93.55%) | 21,163 (93.52%) | 40,053 (93.56%) |  |
| EUR | 2,209 (3.38%) | 686 (3.03%) | 1,523 (3.56%) |  |
| OTR | 734 (1.12%) | 272 (1.20%) | 462 (1.08%) |  |
| DES | 1,280 (1.96%) | 508 (2.24%) | 772 (1.80%) |  |
| Income |  |  |  | 0.016 |
| 0 – 18.000 | 55,769 (85.22%) | 19,181 (84.76%) | 36,588 (85.47%) |  |
| > 18.000 | 9,670 (14.78%) | 3,448 (15.24%) | 6,222 (14.53%) |  |
| Risk of social exclusion | 3,123 (4.77%) | 1,035 (4.57%) | 2,088 (4.88%) | 0.083 |
| *Diagnosis* |  |  |  | 0.016 |
| Atrial fibrillation | 62,702 (95.82%) | 21,624 (95.56%) | 41,078 (95.95%) |  |
| Flutter | 2,737 (4.18%) | 1,005 (4.44%) | 1,732 (4.05%) |  |
| Time since Therapy Initiation |  |  |  | 0.627 |
| 1 – 3 Years | 15,596 (23.83%) | 5,411 (23.91%) | 10,185 (23.79%) |  |
| 3 – 6 Years | 19,003 (29.04%) | 6,611 (29.21%) | 12,392 (28.95%) |  |
| > 6 Years | 30,840 (47.13%) | 10,607 (46.87%) | 20,233 (47.26%) |  |
| *Comorbidities* |  |  |  |  |
| Congestive heart failure | 13,673 (20.89%) | 4,759 (21.03%) | 8,914 (20.82%) | 0.533 |
| Hypertension | 54,370 (83.09%) | 18,817 (83.15%) | 35,553 (83.05%) | 0.731 |
| Diabetes | 25,662 (39.22%) | 8,905 (39.35%) | 16,757 (39.14%) | 0.602 |
| Liver disease | 5,890 (9.00%) | 2,095 (9.26%) | 3,795 (8.86%) | 0.095 |
| Renal disease | 10,558 (16.13%) | 3,684 (16.28%) | 6,874 (16.06%) | 0.461 |
| Previous ischemic stroke or TIA | 9,358 (14.30%) | 3,241 (14.32%) | 6,117 (14.29%) | 0.907 |
| Thromboembolism | 4,577 (6.99%) | 1,609 (7.11%) | 2,968 (6.93%) | 0.397 |
| Hemorrhagic stroke | 446 (0.68%) | 160 (0.71%) | 286 (0.67%) | 0.564 |
| Gastrointestinal bleeding | 4,659 (7.12%) | 1,644 (7.27%) | 3,015 (7.04%) | 0.293 |
| Other bleeding | 21,738 (33.22%) | 7,596 (33.57%) | 14,142 (33.03%) | 0.168 |
| Vascular disease | 12,176 (18.61%) | 4,191 (18.52%) | 7,985 (18.65%) | 0.681 |
| Dementia | 5,547 (8.48%) | 1,916 (8.47%) | 3,631 (8.48%) | 0.949 |
| Depression | 9,808 (14.99%) | 3,403 (15.04%) | 6,405 (14.96%) | 0.794 |
| Cancer | 11,051 (16.89%) | 3,878 (17.14%) | 7,173 (16.76%) | 0.215 |
| Alcohol | 590 (0.90%) | 189 (0.84%) | 401 (0.94%) | 0.191 |
| *Healthcare utilization* |  |  |  |  |
| Hospitalizations | 0.54 (1.18) | 0.54 (1.16) | 0.55 (1.19) | 0.108 |
| ED visits | 1.03 (2.01) | 1.00 (2.00) | 1.04 (2.01) | 0.021 |
| Outpatients visits | 11.89 (7.65) | 12.13 (7.66) | 11.76 (7.64) | 0.000 |
| Specialist visits | 3.36 (4.84) | 3.22 (4.64) | 3.44 (4.95) | 0.000 |
| Cardiology visits | 0.84 (1.20) | 0.83 (1.18) | 0.84 (1.21) | 0.323 |
| Neurologic visits | 0.17 (0.61) | 0.17 (0.60) | 0.17 (0.61) | 0.297 |
| Mental Health visits | 0.11 (0.86) | 0.11 (0.89) | 0.10 (0.84) | 0.531 |
| Social care visits | 0.10 (0.74) | 0.11 (0.74) | 0.10 (0.74) | 0.920 |
| *Medication use* |  |  |  |  |
| NSAID | 6,598 (10.08%) | 2,328 (10.29%) | 4,270 (9.97%) | 0.205 |
| Antiplatelet | 5,651 (8.64%) | 1,903 (8.41%) | 3,748 (8.75%) | 0.135 |
| *Scores* |  |  |  |  |
| CHADS2 score >= 2 | 50,418 (77.05%) | 17,495 (77.31%) | 32,923 (76.90%) | 0.239 |
| CHA2DS2-VASC score >= 2 | 62,257 (95.14%) | 21,567 (95.31%) | 40,690 (95.05%) | 0.143 |
| HAS BLED >=2 | 64,259 (98.20%) | 22,238 (98.27%) | 42,021 (98.16%) | 0.292 |
| HAS BLED >=3 | 56,856 (86.88%) | 19,707 (87.09%) | 37,149 (86.78%) | 0.262 |
